# Insights from an Italian Delphi panel: exploring resistance to first-generation somatostatin receptor ligands and guiding second-line medical therapies in acromegaly management

**DOI:** 10.1007/s40618-024-02386-3

**Published:** 2024-05-29

**Authors:** S. Grottoli, P. Maffei, A. S. Tresoldi, S. Granato, L. Benedan, P. Mariani, A. Giustina

**Affiliations:** 1 Department of Medical Science, Division of Endocrinology, Diabetes and Metabolism, University of Turin, Turin, Italy; 2 Department of Medicine (DIMED), 3rd Medical Clinic, Padua University Hospital, Padua, Italy; 3 Medical Department, Pfizer Italia, Rome, Italy; 4 Università Milano-Bicocca, Milan, Italy; 5 Institute of Endocrine and Metabolic Sciences, Vita-Salute San Raffaele University and IRCCS San Raffaele Hospital, Milan, Italy

**Keywords:** Acromegaly, Somatostatin receptor ligands (SRLs), Pegvisomant, Pasireotide, Type 2 diabetes mellitus, Delphi

## Abstract

**Purpose:**

First-line medical therapy for acromegaly management includes first-generation somatostatin receptor ligands (fgSRLs), but resistance limits their use. Despite international guidelines, the choice of second-line therapy is debated.

**Methods:**

We aim to discuss resistance to fgSRLs, identify second-line therapy determinants and assess glycemia’s impact to provide valuable insights for acromegaly management in clinical practice. A group of Italian endocrinologists expert in the pituitary field participated in a two-round Delphi panel between July and September 2023. The Delphi questionnaire encompassed a total of 75 statements categorized into three sections: resistance to fgSRLs therapy and predictors of response; determinants for the selection of second-line therapy; the role of glycemia in the therapeutic management. The statements were rated on a 6-point Likert scale.

**Results:**

Fifty-nine (79%) statements reached a consensus. IGF-1 levels resulted central for evaluating resistance to fgSRLs, that should be defined considering also symptomatic clinical response, degree of tumor shrinkage and complications, using clinician- and patient-reported outcome tools available. Factors to be evaluated for the choice of second-line medical therapy are hyperglycemia—that should be managed as in non-acromegalic patients—tumor remnant, resistant headache and compliance. Costs do not represent a main determinant in the choice of second-line medical treatment.

**Conclusion:**

The experts agreed on a holistic management approach to acromegaly. It is therefore necessary to choose currently available highly effective second-line medical treatment (pegvisomant and pasireotide) based on the characteristics of the patients.

**Supplementary Information:**

The online version contains supplementary material available at 10.1007/s40618-024-02386-3.

## Introduction

Acromegaly is a chronic, disabling, rare endocrine disorder characterized by the excessive production of the growth hormone (GH) and insulin-like growth factor 1 (IGF-1) [1–3]. Systemic long-term complications of acromegaly include cardiovascular and metabolic complications, respiratory disorders, arthropathy, and depression, with an impact on quality of life (QoL); it is overall associated with potentially life-threatening effects [1, 4].

First-line multimodal management of acromegalic patients includes surgical interventions aiming to remove the pituitary adenoma responsible for excessive GH overproduction. Subsequent medical therapy aims to control tumor growth, inhibit GH hypersecretion, normalize IGF-1 levels, and reduce the burden of comorbidities. Finally, radiotherapy is used when surgery and medical treatments are not curative or have significant risks [5, 6].

The first-line medical treatment includes first-generation somatostatin receptor ligands (fgSRLs) such as octreotide and lanreotide [5, 6], although resistance to fgSRLs is common in clinical practice and is reported in up to 66% of patients [7, 8]. Several predictors of resistance have been described and include the patients’ gender, age, initial GH and IGF-1 levels, tumor volume, tumor hyperintensity on T2-weighted magnetic resonance imaging, and the expression of somatostatin receptor subtypes [7]. For patients who are not controlled while on fgSRLs, second-line therapies are available to achieve biochemical control of the disease. These include GH receptor antagonist pegvisomant (PEGV) and second-generation SRLs pasireotide (PASI) [5, 6, 9], while dopamine agonist cabergoline is only used in selected cases [10]. Treatment with PASI has been associated with a dysregulation of glucose metabolism and the onset of diabetes [11], while treatment with PEGV has been associated with an improvement in glycemic control [12].

Despite several international consensus and guidelines provide recommendations on the therapeutic algorithm for the management of acromegaly based on different patient’s characteristics [5, 11], a specific document providing indication on the current management of acromegalic patients in clinical practice in Italy is missing. To this end, Italian endocrinologists expert in acromegaly’s management were involved in a Delphi panel to obtain indications on the definition of resistance to fgSRLs, the biochemical and clinical determinants to drive the second-line therapy and the impact of glycemia in the therapeutic approach.

## Methods

### The Delphi method

The Delphi method involves multiple rounds of anonymous surveys that allow experts to provide feedback and revise their responses based on the collective insights of the group. The method facilitates the achievement of a consensus [13].

The fundamental prerequisites of a high-quality Delphi study are anonymity, iteration, controlled feedback, and statistical stability of consensus [14]. The details of Delphi methods and statements’ definition are reported in Supplementary material.

### Participant selection

For Delphi studies it is crucial to assemble a panel of experts, essential for generating valuable insights and reliable answers [15, 16]. Participants were each individually selected by the Steering Committee (AG, Silvia G, and Pietro M) based on their qualifications and expertise in the management of acromegaly, to form a diverse and knowledgeable panel that ensures a comprehensive coverage of the endocrinology centers nationwide. Selected panelists belonged to referral centers for neuroendocrinology and had long-term experience in acromegaly management. In the invitation email, the panelists were provided with an explanation of the nature and the aim of the project. The invited endocrinologists were not involved in creating the survey, but only to express their opinion to the statements.

### Questionnaires

The present questionnaire encompassed a total of 75 statements categorized into three sections:

- Incidence, definition of resistance to fgSRLs therapy, and predictors of response (21 items)
- Biochemical and clinical determinants for the selection of second-line therapy (29 items)
- The role of glycemia in the therapeutic management of the patient (25 items).

Participants provided ratings on a 6-point Likert scale, ranging from strong disagreement to full agreement. The survey was available for completion through a dedicated online platform (SurveyMonkey®) over 10 days in July 2023. The results of the first round were discussed by the Steering Committee and, as per Delphi method, statements that did not reach consensus threshold in the first round were resubmitted for further consideration in a second round, on the same platform, for another 10 days in September 2023. A total of 28 statements were resubmitted to the panelists. Slight revisions to two statements were made to eliminate any potential ambiguities or misinterpretations (Supplementary material).

In 2017, a similar Delphi questionnaire was proposed to a panel of experts. That study was not published, but the results were made available to the authors for discussion and comparison with the current results, taking into consideration that many statements had to be changed to be in line with the most recent clinical development. The 2017 Delphi encompassed 61 statements, subdivided in the following topics: SRLs therapy, resistance to SRLs, second-line choice, evaluation of hyperglycemia and comorbidities in patients with acromegaly. The questionnaire was evaluated on 5-point Likert scale. The complete text of the 2017 Delphi questionnaire is reported in the Supplementary material.

### Data analysis

The data were analyzed by descriptive statistics. Clear a priori criteria were established to define the conditions under which the Delphi study’s conclusions would be considered reached [17]. Consensus was to be deemed achieved when a minimum agreement threshold of 67% was met. The same criteria were applied to the Delphi questionnaire performed in 2017, albeit with some minor differences in the scale used (Supplementary material).

## Results

A total of 66 endocrinologists were involved in the Delphi panel; during the first round, 54 experts submitted a response to at least one statement, and 48 completed the full questionnaire. The second round involved the 53 experts who responded to at least one statement of the first round (one of the panelists agreed not to proceed further) and 36 full responses were collected. Overall, 59 out of 75 (79%) statements reached a consensus, either in agreement or disagreement. In the 16 cases where the agreement threshold was not met, the values from the first round, which had the highest number of respondents, were considered as the final results.

### Incidence, definition of resistance to fgSRLs therapy, and predictors of response

The first 21 statements explored the resistance to fgSRLs in the clinical management of acromegaly, with 17 reaching a consensus (Table 1). The highest level of agreement was reached for the lack of somatostatin receptors on adenoma and genetic predisposition as characteristic predicting resistance to fgSRLs (96% and 92%, respectively), and for IGF-1 levels being central for evaluating resistance (89% agreement). Seventy-eight percent of experts stated that IGF-1 alone should be considered in case of discrepancy with GH values, and that multiple measurements over time are needed in case of moderately increased values (83% agreement). Beside IGF-1, GH levels should also be considered to monitor fgSRL resistance (75% agreement for considering both values) but it should not be used alone (71% disagreement). More frequent assessment of IGF-1/GH levels is pivotal for patients not achieving disease control (88% of agreement); slightly elevated IGF-1/GH levels are tolerated for patients who underwent radiosurgery (75% agreement), but not for those over 50 years that have been recently diagnosed (76% disagreement). No agreement was achieved in specific statements exploring results in discrepant patients.

**Table 1:** List of all the statements related to incidence, definition of resistance to fgSRLs therapy, and predictors of response, consensus reached, percentage of consensus, and number of responders for each statement

***Incidence, definition of resistance to therapy with first-generation somatostatin analogs (fgSRLs), and predictors of response***				
		Consensus	% Consensus^a^	N
1. I believe that, in evaluating the condition of biochemical resistance to therapy with fgSRLs (octreotide-lanreotide):	1.1. IGF-1 levels represent the “gold standard” for assessing resistance to fgSRLs therapy	R1: Agreement	**89%**	54
	1.2. To assess resistance to fgSRLs therapy, it is necessary to repeat the measurement of IGF-1 multiple times over time, especially when its increase is moderate	R1: Agreement	**83%**	54
	1.3. IGF-1 levels within the normal range represent an important goal of therapy, but they can be considered satisfactory even if they remain slightly above the normal range 1*.*3 *Second round: IGF-1 levels within the normal range represent an important goal of therapy, but they can be considered satisfactory even if they remain slightly above the normal range (1.0 – 1.3 x ULN)*	Not reached	55%	53
	1.4. Both IGF-1 and GH levels are essential in defining a condition of resistance to fgSRLs therapy	R1: Agreement	**75%**	52
	1.5. In my experience, IGF-1 measurements exhibit limited or inconsistent reliability, and therefore, GH levels should continue to be used to decide whether the patient is resistant to fgSRLs therapy	R1: Disagreement	**71%**	52
2. I believe that to contextualize the role of biochemical parameters in assessing resistance to fgSRLs therapy (octreotide-lanreotide):	2.1. Slightly elevated IGF-1 levels (1.0-1.3 x ULN) during fgSRLs therapy are tolerable in a patient over 50 years old with a recent diagnosis, as in their case, acromegaly may be less aggressive	R2: Disagreement	**76%**	37
	2.2. Slightly elevated IGF-1 levels (1.0-1.3 x ULN) during fgSRLs therapy are tolerable in a patient who has already undergone stereotactic radiosurgery, as a progressive improvement in disease control is expected	R1: Agreement	**75%**	51
	2.3. In patients who do not achieve optimal disease control during fgSRLs therapy, GH and IGF-1 measurements, as well as clinical assessments, should be repeated more frequently	R1: Agreement	**88%**	51
	2.4. In case of discrepancy between normalization of IGF-1 and GH, the assessment of resistance to fgSRLs should be based only on IGF-1	R1: Agreement	**78%**	51
	2.5. In case of discrepancy between normalization of IGF-1 and GH, to define acromegaly as controlled, both values need to fall within the target range *2.5. Second round: In case of discrepancy between normalization of IGF-1 and GH, the disease cannot be considered controlled as I believe control is achieved only when both parameters are normalized*	Not reached	57%	51
	2.6. In a patient with IGF-1 levels between 1.0-1.3 x ULN, to define the patient as controlled, a random GH value < 1.0 ng/ml is necessary	Not reached	56%	50
3. I believe that the biochemical definition of resistance to fgSRL therapy (octreotide-lanreotide) should be integrated with:	3.1. The symptomatic clinical response	R1: Agreement	**86%**	50
	3.2. The degree of tumor shrinkage lower than 20% of the initial mass	R2: Agreement	**69%**	36
	3.3. The clinical response in terms of complications	R1: Agreement	**72%**	50
	3.4. The holistic evaluation using appropriate tools (ACRODAT^®^ / SAGIT^®^)	R1: Agreement	**76%**	50
	3.5. The presence of treatment-related side effects such as hyperglycemia	R1: Disagreement	**68%**	50
4. Resistance to fgSRLs therapy (octreotide-lanreotide) in first-line medical treatment can be predicted:	4.1. In a young patient with familiar history and positive genetics	R1: Agreement	**92%**	49
	4.2. In a patient with a significant residual adenomatous volume on post-operative magnetic resonance imaging	R2: Disagreement	**69%**	36
	4.3. In a patient with elevated post-surgical GH/IGF-1 levels	Not reached	59%	49
	4.4. In a patient with an adenoma that does not express SSTR 2-5 on post-operative histological examination	R1: Agreement	**96%**	49
	4.5. In a patient with molecular markers of aggressiveness on post-operative histological examination	R1: Agreement	**88%**	49

Percentages reaching the minum agreement threshold are in bold

^**a**^In case a consensus was not reached during the two rounds, the percentage reported is the percentage of agreement achieved in the first round, where the highest number of opinions were received

*R1* round 1, *R2* round 2

A good level of agreement was reached for all statements exploring the integration of different parameters (symptomatic clinical response, degree of tumor shrinkage, complications, use of clinician-reported outcomes [CROs]/patient-reported outcomes [PROs] tools) in the definition of fgSRLs resistance (range 69–86%), while hyperglycemia is not seen as a parameter to be integrated for the biochemical definition of resistance (68% disagreement).

### Biochemical and clinical determinants for the choice of second-line medical therapy

Statements from 22 to 50 analyzed the determinants influencing the choice of second-line medical therapy, with 23 reaching a consensus (Table 2). The highest level of agreement was for cost not influencing the choice of fgSRLs therapy (90%). PEGV monotherapy or a combination of fgSRLs + PEGV are a second-line choice for 88% of experts, while PASI is for 86% of them.

**Table 2:** List of all the statements related to biochemical and clinical determinants for the choice of second-line therapy, consensus reached, percentage of consensus, and number of responders for each statement

***Biochemical and clinical determinants for the choice of second-line therapy***				
		Consensus	% Consensus^a^	N
5. In a biochemically uncontrolled patient on fgSRLs (octreotide-lanreotide) therapy, I consider as a following possible therapeutic step:	5.1. A combination therapy of fgSRLs (octreotide–lanreotide) + cabergoline	Not reached	53%	49
	5.2. A pegvisomant monotherapy	R1: Agreement	**88%**	49
	5.3. A combination therapy of fgSRLs (octreotide–lanreotide) + pegvisomant	R1: Agreement	**88%**	49
	5.4. A second-generation SRL (pasireotide) therapy	R1: Agreement	**86%**	49
6. I believe that the element that motivates me to the shift to pegvisomant monotherapy in a patient resistant to first- and second-generation SRLs (octreotide-lanreotide-pasireotide) can be represented by:	6.1. Slightly elevated IGF-1 levels (1.3-1.5 x ULN) during first- or second-generation SRLs therapy	R1: Agreement	**69%**	49
	6.2. Elevated IGF-1 levels (> 1.5 x ULN) during first- or second-generation SRLs therapy	R1: Agreement	**86%**	49
	6.3. The presence of diabetes mellitus	R1: Agreement	**86%**	49
	6.4. Cardiovascular complications	R2: Disagreement	**75%**	36
	6.5. A history of radiotherapy	R1: Agreement	**71%**	49
7. In the decision to shift to pegvisomant monotherapy in a patient resistant to first- and second-generation SRLs (octreotide-lanreotide-pasireotide), the following can be a cause for concern:	7.1. The regrowth of the pituitary adenoma upon SRLs discontinuation	R1: Agreement	**69%**	48
	7.2. The therapy costs	R1: Disagreement	**79%**	48
	7.3. The patient's compliance when transitioning to daily therapy with pegvisomant	R1: Agreement	**67%**	48
	7.4. The recurrence of headache in a patient for whom this symptom was responsive to SRLs therapy	R1: Agreement	**83%**	48
	7.5. I do not see any specific reasons for concern	R2: Agreement	**72%**	36
8. I believe that second-generation SRL (pasireotide) can be used:	8.1. Only after treatment with pegvisomant monotherapy or in combination with fgSRLs (octreotide–lanreotide)	R1: Disagreement	**81%**	48
	8.2. In the presence of severe headache regardless of the response to other pharmacological therapies	R2: Agreement	**69%**	36
	8.3. With no concerns if there is no history of diabetes mellitus	R1: Agreement	**73%**	48
	8.4. If diabetes mellitus is controlled with diet alone, only after treatment with metformin	Not reached	52%	48
	8.5. Without any issues if diabetes mellitus is already being treated with GLP-1 receptor agonists	Not reached	52%	48
9. I believe that the costs of pharmacological therapy can influence my choice of treatment with:	9.1. A FgSRLs (octreotide–lanreotide) therapy	R1: Disagreement	**90%**	48
	9.2. A combination therapy of fgSRLs (octreotide–lanreotide) + pegvisomant	R1: Disagreement	**69%**	48
	9.3. A pegvisomant monotherapy	R1: Disagreement	**79%**	48
	9.4. A second-generation SRL (pasireotide) therapy	R1: Disagreement	**83%**	48
	9.5. A combination therapy of second-generation SRLs (pasireotide) + pegvisomant	Not reached	44%	48
10. Considering the patient's compliance and adherence, I would adopt the following therapeutic strategy:	10.1. A combination therapy of fgSRLs (octreotide–lanreotide) + cabergoline	Not reached	54%	48
	10.2. A second-generation SRLs (pasireotide) therapy	R1: Agreement	**85%**	48
	10.3. A pegvisomant monotherapy (even if not daily)	R1: Agreement	**83%**	48
	10.4. A combination therapy of pegvisomant + fgSRLs (octreotide–lanreotide)	R1: Agreement	**79%**	48
	10.5. A combination therapy of pegvisomant + second-generation SRL (pasireotide)	Not reached	58%	48

Percentages reaching the minum agreement threshold are in bold

^**a**^In case a consensus was not reached during the two rounds, the percentage of agreement is given with respect to the first round, where the highest number of opinions were received

*R1* round 1, *R2* round 2

Overall, the panel reached a consensus on the indications for the shift to PEGV monotherapy and the related possible causes of concern. For instance, the presence of type 2 diabetes mellitus (T2DM) and IGF-1 levels > 1.5 × ULN motivate to PEGV shift after SRLs resistance (86% each), and the recurrence of previously controlled headaches is a concern for 83% of the panelists.

PASI can be used with no concern in the absence of T2DM and in case of severe headache regardless of the response to other therapies (73% and 69% agreement, respectively); previous therapy with PEGV (either as a monotherapy or combination with fgSRLs) is not a prerequisite for switching to this therapy (81% disagreement). Costs influence the therapy choice only in case of PASI + PEGV, which did not reach an agreement. Considering patient’s compliance and adherence, 85% of experts would choose PASI, 83% PEGV as monotherapy and 79% a combination of fgSRLs + PEGV.

Statements not reaching an agreement concerned fgSRLs and cabergoline combination therapy, PASI and PEGV combination therapy, and the management of T2DM in a patient starting PASI therapy.

### Role of blood glucose levels in the therapeutic management of the patient

Twenty-five statements investigated the influence of blood glucose levels on the choice of treatment, with 19 reaching a consensus (Table 3). The panel disagreed on impaired fasting glucose (IFG) being a contraindication for the use of PASI, or a strict indication for switching to PEGV monotherapy in young acromegalic patients (83% and 75%, respectively). A good level of consent was achieved for the statements exploring the management of hyperglycemia, with the highest consensus on target HbA1c being age-dependent (94%). Ninety-four percent of panelists also agreed that a controlled patient who develops T2DM should be maintained on fgSRLs; 90% disagreed in switching to PEGV monotherapy in this condition, which should be initiated only if hyperglycemia control is not achieved (69% agreement). In case of resistance to fgSRLs, experts did not agree on PASI therapy to be started only in association with PEGV (92% disagreement), while for 67% HbA1c levels guide the choice of the second-line treatment.

**Table 3:** List of all the statements related to biochemical and clinical determinants for the choice of second-line therapy, consensus reached, percentage of consensus, and number of responders for each statement

***Role of blood glucose levels in the therapeutic management of the patient***				
		Consensus	% Consensus^a^	N
11. The presence of impaired fasting glucose (IFG) in a young acromegalic patient resistant to fgSRLs therapy (octreotide-lanreotide):	11.1. Advises against the use of second-generation SRL (pasireotide)	R2: Disagreement	**83%**	36
	11.2. Is not a crucial factor in the choice of therapy	Not reached	48%	48
	11.3. Is an indication for the addition of pegvisomant	Not reached	56%	48
	11.4. Recommends discontinuation of fgSRLs therapy and initiation of pegvisomant monotherapy	R2: Disagreement	**75%**	36
	11.5. Impose the initiation of antidiabetic treatment and consequently the start of a therapy with second-generation SRL (pasireotide) to replace the fgSRLs	R1: Disagreement	**77%**	48
12. I believe that in an acromegalic patient who develops hyperglycemia:	12.1. The specific acromegaly therapy should be modified before addressing the diabetes issue	R1: Disagreement	**73%**	48
	12.2. An HbA1c level below 7.0% should be reached and a specific diabetes treatment, depending on the molecule used for acromegaly treatment, should be started	R1: Agreement	**85%**	48
	12.3. The achievement of an HbA1c level below 6.5% should be obtained through intensive diabetes treatment	R2: Agreement	**81%**	36
	12.4. The HbA1c target level depends on the patient's age	R1: Agreement	**94%**	48
	12.5. Patient should be referred to the designated Diabetology Center	Not reached	40%	48
	12.6. The glycemic control target should be assessed based on other cardiovascular risk factors (blood pressure, lipid profile, body weight, OSAS...)	R1: Agreement	**90%**	48
13. In a well-controlled acromegalic patient undergoing fgSRLs (octreotide-lanreotide) therapy who develops diabetes mellitus, I believe that:	13.1. FgSRLs (octreotide–lanreotide) dose should be reduced or the SRLs therapy should be discontinued, and treatment with pegvisomant should be initiated	R1: Disagreement	**90%**	48
	13.2. FgSRLs (octreotide–lanreotide) therapy should be maintained, and an anti-hyperglycemic pharmacological treatment should be initiated	R1: Agreement	**94%**	48
	13.3. FgSRLs (octreotide–lanreotide) therapy should be discontinued and treatment with pegvisomant should be initiated as monotherapy only if hyperglycemia cannot be managed with antidiabetic medications	R2: Agreement	**69%**	36
	13.4. The first-line treatment for diabetes should be DPP-4 inhibitors or GLP-1 receptor agonists	Not reached	52%	48
	13.5. Diabetes should be treated as in any other 'non-acromegalic' patient	R1: Agreement	**67%**	48
14. In an acromegalic patient resistant to first-generation SLRs therapy (octreotide - lanreotide) who develops diabetes mellitus:	14.1. The choice of second-line therapy depends on the HbA1c levels	R1: Agreement	**67%**	48
	14.2. Diabetes does not influence the therapeutic choice; GH/IGF-1 levels affect glycemic metabolism, so my primary goal remains the hormonal normalization of acromegaly	Not reached	48%	48
	14.3. The addition of pegvisomant is necessary	Not reached	52%	48
	14.4. Treatment with second-generation SRL (pasireotide) can be initiated only in combination with pegvisomant	R1: Disagreement	**92%**	48
15. In a 35-year-old patient with a history of gestational diabetes mellitus (GDM) and a GH-secreting macroadenoma resistant to monthly octreotide LAR 30 mg, I consider the following pharmacological treatment as appropriate:	15.1. The discontinuation of octreotide LAR therapy and the initiation of a second-generation SSA (pasireotide) therapy, also for its tumor-shrinking effect; the GDM is not a current concern	R2: Agreement	**75%**	36
	15.2. A combination therapy of octreotide LAR with pegvisomant to achieve hormonal normalization and control the tumor mass	R1: Agreement	**75%**	48
	15.3. The discontinuation of octreotide LAR therapy and the initiation of pegvisomant monotherapy; it is effective hormonally and has a positive impact on glycemic metabolism	R2: Disagreement	**81%**	36
	15.4. No pharmacological therapy and consideration of neurosurgery	R1: Agreement	**90%**	48
	15.5. The continuation of therapy with octreotide LAR and the addition of cabergoline; the dopamine agonist enhances the effect of octreotide most patients, and has a favorable impact on glycemic metabolism	R1: Disagreement	**67%**	48

Percentages reaching the minum agreement threshold are in bold

^**a**^In case a consensus was not reached during the two rounds, the percentage of agreement is given with respect to the first round, where the highest number of opinions were received

*R1* round 1, *R2* round 2

When presented with a clinical case scenario of a woman with previous gestational diabetes mellitus (GDM) and a GH-secreting macroadenoma resistant to fgSRLs, experts agreed on neurosurgery as the next therapeutic step (90%), followed by association of fgSRLs + PEGV (75%) or a switch to PASI monotherapy (75%), while a switch to PEGV monotherapy or fgSRL + cabergoline association therapy were not deemed adequate options (81% and 67% disagreement respectively).

Six statements did not reach consensus: IFG guiding the choice of therapy or being an indication for the addition of PEGV, the need to refer acromegalic diabetic patients to a Diabetology center, the consideration on the use of DPP-4 inhibitors or GLP-1 receptor agonists in a controlled patient, and the influence of T2DM in the therapy choice in case of fgSRL resistance or the necessity to add PEGV in such patients.

### Dynamic comparison between 2017 and 2023 Delphi panels

**Fig. 1:**
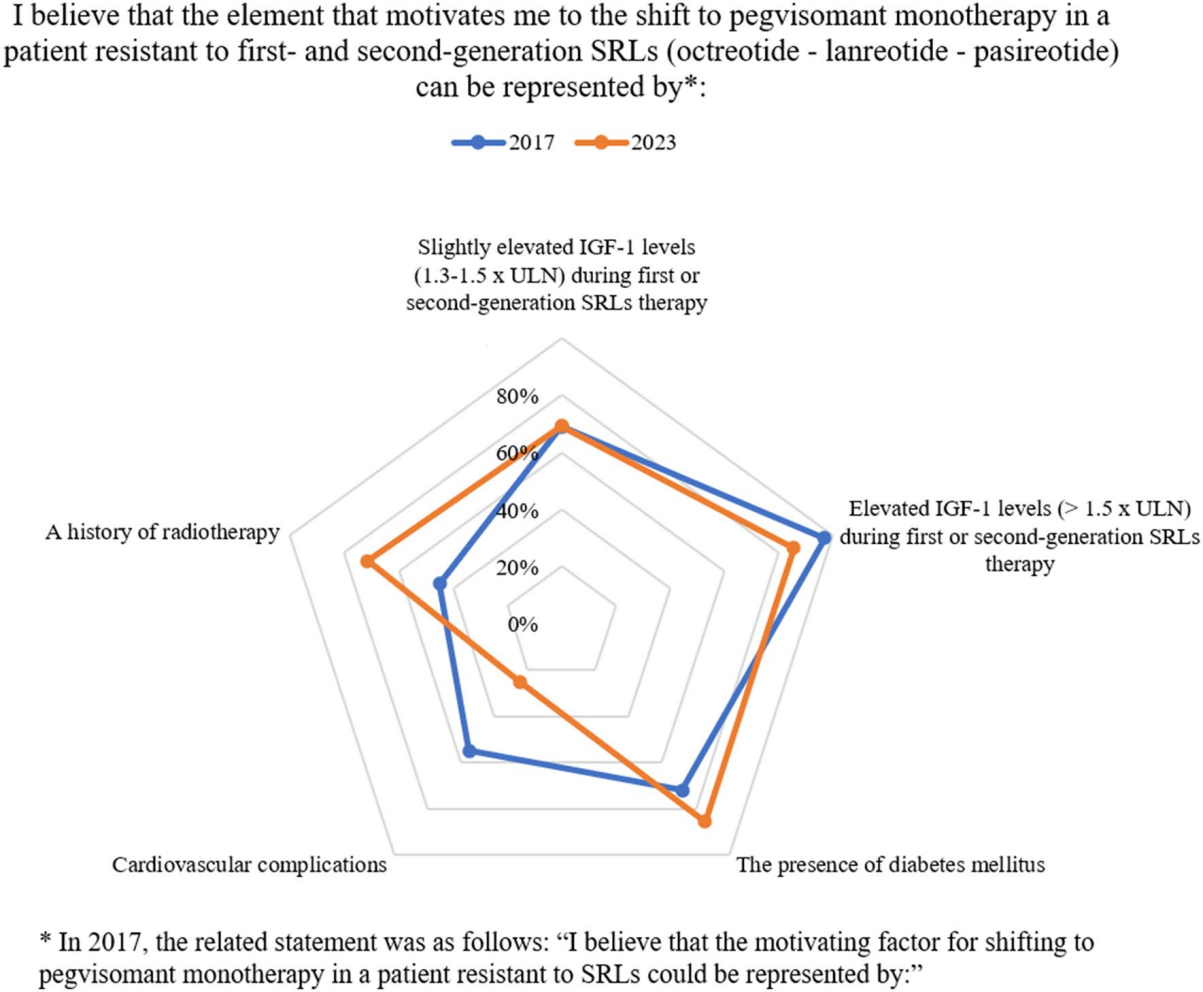
Dynamic comparison–Sect. 2, statement 6-% of consensus

**Fig. 2:**
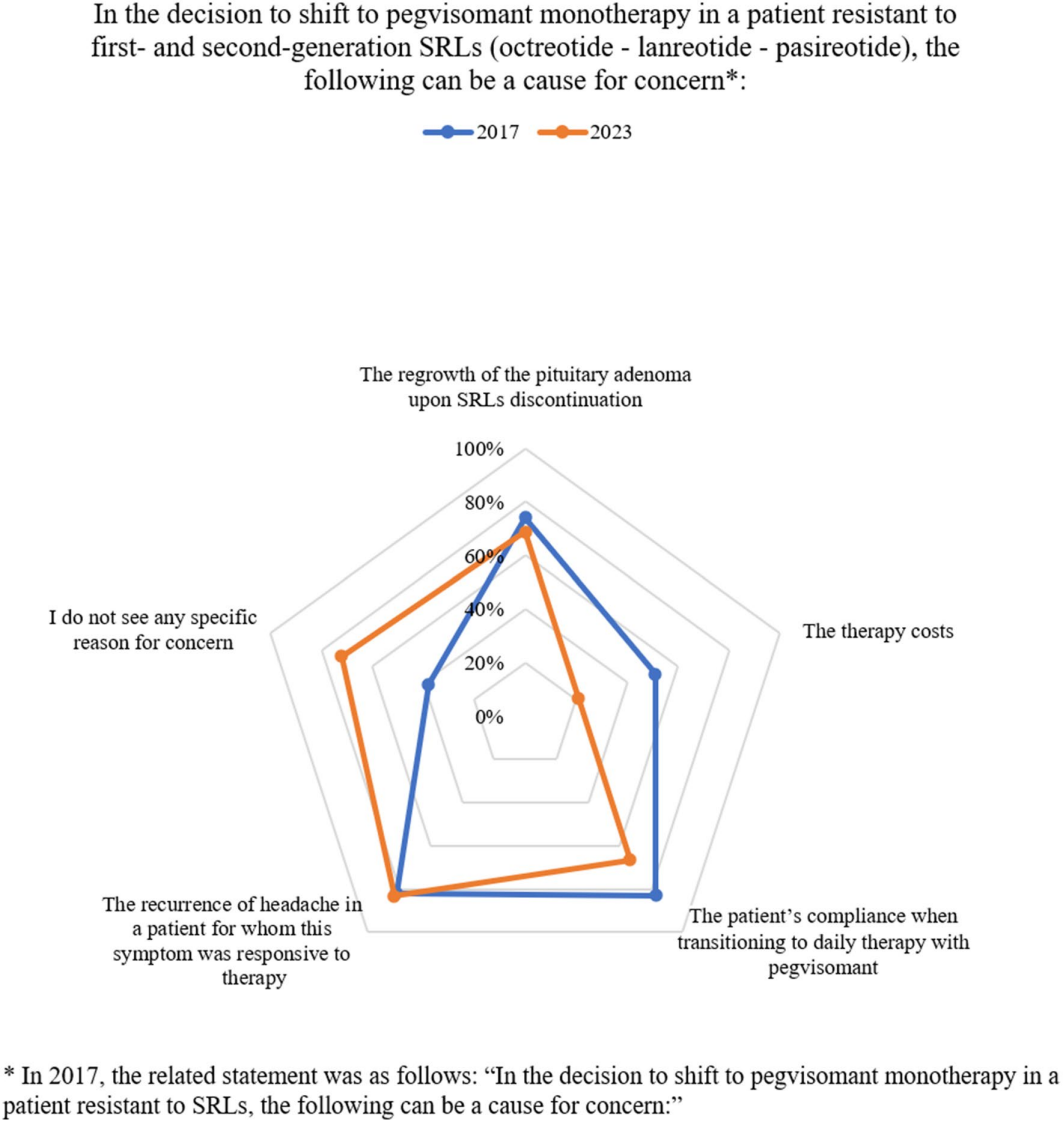
Dynamic comparison–Sect. 2, statement 7-% of consensus

A dynamic comparison with the findings of the prior 2017 Delphi study was performed, although many statements had to be changed to be in line with the most recent clinical development. The previous study involved 78 endocrinologists. As shown in Table 4, for most of the comparable statements, a significant overlap of the responses was noticed, indicating a certain level of consistency in the viewpoints of clinicians regarding the various issues raised. However, discrepancies on some points were highlighted as shown in Figs. 1 and 2.

**Table 4:** Comparison of the percentage of consensus between the 2017 Delphi and the current study

**Statement**		**2017**	**2023**
*Delphi 2017: I believe that in the evaluation of resistance to somatostatin receptor ligands (SRLs):* Delphi 2023: I believe that in evaluating the condition of biochemical resistance to therapy with fgSRLs (octreotide-lanreotide):	2017: IGF-1 levels represent the "gold standard" for assessing resistance to SRLs 2023: IGF-1 levels represent the "gold standard" for assessing resistance to fgSRLs therapy	A: **89%**	A: **89%**
	2017: To assess resistance to SRLs, it is necessary to repeat the IGF-1 dosage several times over time, especially when its increase is moderate 2023: To assess resistance to fgSRLs therapy, it is necessary to repeat the measurement of IGF-1 multiple times over time, especially when its increase is moderate	A: **91%**	A: **83%**
	2017: IGF-1 levels within the normal range represent an important goal of therapy, but they can be considered satisfactory even if they remain slightly above normal 2023: IGF-1 levels within the normal range represent an important goal of therapy, but they can be considered satisfactory even if they remain slightly above the normal range	A: 61%^a^	A: 55%^a^
	2017: Both IGF-1 and GH levels are essential in defining a condition of resistance to SRLs 2023: Both IGF-1 and GH levels are essential in defining a condition of resistance to fgSRLs therapy	A: **83%**	A: **75%**
	2017: In my experience, IGF-1 measurements exhibit limited or inconsistent reliability, and therefore GH levels should continue to be used to decide whether the patient is resistant to SRLs therapy 2023: In my experience, IGF-1 measurements exhibit limited or inconsistent reliability, and therefore, GH levels should continue to be used to decide whether the patient is resistant to fgSRLs therapy	D: **67%**	D: **75%**
*Delphi 2017: I believe that to contextualize the role of biochemical parameters in assessing resistance to SRLs:* Delphi 2023: I believe that to contextualize the role of biochemical parameters in assessing resistance to fgSRL therapy (octreotide - lanreotide):	2017: Slightly increased IGF-1 levels (1.0-1.5 x ULN) during SRLs therapy are tolerable in a patient over 50 with a recent diagnosis, as in their case, acromegaly could be less aggressive 2023: Slightly elevated IGF-1 levels (1.0-1.3 x ULN) during fgSRLs therapy are tolerable in a patient over 50 years old with a recent diagnosis, as in their case, acromegaly may be less aggressive	D: **68%**	D:**76%**
	2017: Slightly increased IGF-1 levels (1.0-1.5 x ULN) during SRLs therapy are tolerable in a patient who has already undergone stereotactic radiosurgery as a progressive improvement in disease control is expected 2023: Slightly elevated IGF-1 levels (1.0-1.3 x ULN) during fgSRLs therapy are tolerable in a patient who has already undergone stereotactic radiosurgery, as a progressive improvement in disease control is expected	A: **87%**	A: **75%**
	2017: In patients who do not achieve optimal disease control during SRLs therapy, GH and IGF-1 measurements, as well as clinical assessments should be repeated more frequently 2023: In patients who do not achieve optimal disease control during fgSRLs therapy, GH and IGF-1 measurements, as well as clinical assessments should be repeated more frequently	A: **87%**	A: **88%**
*Delphi 2017: I believe that the motivating factor for shifting to pegvisomant monotherapy in a patient resistant to SRLs could be represented by:* Delphi 2023: I believe that the element that motivates me to the shift to pegvisomant monotherapy in a patient resistant to first- and second-generation SRLs (octreotide-lanreotide-pasireotide) can be represented by:	2017: Elevated IGF-1 in the range 1.1-1.5 x ULN (during SRLs therapy) 2023: Slightly elevated IGF-1 levels (1.3-1.5 x ULN) during first or second-generation SRLs therapy	A: **69%**	A: **69%**
	2017: Elevated IGF-1 > 1.5 x ULN (during SRLs therapy) 2023: Elevated IGF-1 levels (> 1.5 x ULN) during first- or second-generation SRLs therapy	A: **97%**	A: **86%**
	2017: The presence of diabetes 2023: The presence of diabetes mellitus	A: **72%**	A: **86%**
	2017: Cardiovascular complications 2023: Cardiovascular complications	D: 45%^a^	D: **75%**
	2017: A history of radiotherapy 2023: A history of radiotherapy	A: 45%^a^	A: **71%**
*Delphi 2017: In the decision to shift to pegvisomant monotherapy in a patient resistant to SRLs, the following can be a cause for concern:* Delphi 2023: In the decision to shift to pegvisomant monotherapy in a patient resistant to first- and second-generation SRLs (octreotide-lanreotide-pasireotide), the following can be a cause for concern:	2017: The regrowth of the pituitary adenoma upon SRLs discontinuation 2023: The regrowth of the pituitary adenoma upon SRLs discontinuation	A: **74%**	A: **69%**
	2017: The therapy costs 2023: The therapy costs	D: 49%^a^	D: **79%**
	2017: The patient's compliance when transitioning to daily therapy 2023: The patient's compliance when transitioning to daily therapy with pegvisomant	A: **83%**	A: **67%**
	2017: The recurrence of headache in a patient for whom this symptom was responsive to SRLs therapy 2023: The recurrence of headache in a patient for whom this symptom was responsive to SRLs therapy	A: **82%**	A: **83%**
	2017: I do not see any specific reason for concern 2023: I do not see any specific reason for concern	A: 38%^a^	A: **72%**
*Delphi 2017: I believe that the following therapeutic goals should be pursued in the acromegalic patient who develops hyperglycemia, with the following monitoring methods:* Delphi 2023: I believe that in acromegalic patients who develops hyperglycemia:	2017: An HbA1c level below 7.0% with the same frequency and modalities of the non-acromegalic diabetic patient (e.g., every 3-6 months based on the degree of compensation) 2023: An HbA1c level below 7.0% should be reached and a specific diabetes treatment, depending on the molecule used for acromegaly treatment, should be started	A: **92%**	A: **85%**
	2017: A more ambitious HbA1c level (e.g., less than 6.5%) with greater frequency and use of glycemic self-monitoring compared to non-acromegalic diabetic patients 2023: The achievement of an HbA1c level below 6.5% should be obtained through intensive diabetes treatment	D: **76%**	A: **81%**
*Delphi 2017: In a well-controlled acromegalic patient undergoing therapy with SRLs who develops diabetes mellitus, I believe that:* Delphi 2023: In a well-controlled acromegalic patient undergoing fgSRLs (octreotide-lanreotide) therapy who develops diabetes mellitus, I believe that:	2017:SRLs dose should be reduced or the SRLs therapy should be discontinued, and treatment with pegvisomant should be initiated 2023:FgSRLs (octreotide–lanreotide) dose should be reduced or the SRLs therapy should be discontinued, and treatment with pegvisomant should be initiated	D: **72%**	D: **90%**
	2017:SRLs therapy should be maintained, and an anti-hyperglycemic pharmacological treatment should be initiated 2023:FgSRLs (octreotide–lanreotide) therapy should be maintained, and an anti-hyperglycemic pharmacological treatment should be initiated	A: **87%**	A: **94%**
	2017: SRLs therapy should be discontinued, and treatment with pegvisomant should be initiated only if hyperglycemia cannot be managed with antidiabetic medications 2023: FgSRLs (octreotide–lanreotide) therapy should be discontinued, and treatment with pegvisomant should be initiated as monotherapy only if hyperglycemia cannot be managed with antidiabetic medications	A: 65%^a^	A: **69%**

Percentages reaching the minum agreement threshold are in bold

^a^these statements did not achieved consensus

*A* agreement, *D* disagreement

## Discussion

In this work, by means of the Delphi methodology, a panel of Italian endocrinologists assessed factors determining fgSRL resistance, second-line medical treatment [18–20], and T2DM’s role in patient management. IGF-1 is unequivocally established as the primary parameter for monitoring treatment efficacy in clinical practice. The panel revealed low confidence and interest in the use of cabergoline in second-line, while all the other choices are considered according to patient’s condition. Hyperglycemia is an important issue, and it could influence the choice of therapy, especially in certain categories of patients.

### Incidence, definition of resistance to therapy with fgSRLs, and predictors of response

Although both GH and IGF-1 levels are central in defining resistance to treatment (statement 1.4), the panel acknowledges that IGF-1 is the “gold standard” (statement 1.1). The role of IGF-1 is consolidated [21, 22], as it was similarly recognized as determinant also in 2017. On the other hand, the agreement on the role of GH results weaker compared to 2017 (75% vs 83% in 2023 vs 2017, respectively), in line with recent consensus [3].

In case of discrepant IGF-1/GH values, resistance is primarily determined by the levels of IGF-1(statement 2.4). Indeed, in one third of acromegalic patients, GH levels may give discrepant information compared to IGF-1, likely due to assay or cut-off issues, GHR polymorphism, timing of post-surgical assessment, or different biological significance of the two parameters [11, 23–25]. However, no consensus was reached on some statements on the definition of acromegaly under control when IGF-1 and GH levels show different responses to therapies (statements 2.5 and 2.6). In fact, for some of the experts, normalization of GH should be a clinically relevant goal irrespective of IGF-1 normalization, possibly because of the fear of a prospective increase of IGF-1 levels in the absence of GH control; however, this concept has not been included in new Consensus recommendations [3]. Nevertheless, the responses suggest potentially significant implications for clinical practice, since it could involve treatment intensification or increased follow-up even in cases of IGF-1 normalization. This might also indicate a preference for a different second-line drug that lowers GH rather than IGF-1 in cases of discrepancies.

Due to IGF-1 assay variability, particularly in mildly elevated cases, experts stress the importance of repeated evaluations over time before confirming resistance (statement 1.2). This aligns with the 2017 Delphi, albeit with lower agreement (91% in 2017 and 83% in 2023). No consensus was reached regarding whether slightly elevated IGF-1 levels should be considered an indication of effective treatment (statement 1.3). Indeed, not all the guidelines suggest as acceptable target levels up to 1.3 × ULN [4, 11]. A less ambitious target in patients who underwent radiotherapy is acceptable since progressive improvement of disease activity’s control is expected (statement 2.2). However, IGF-1 levels slightly above the normal range are not considered an appropriate target in elderly patients with a recent diagnosis, where the disease could be less aggressive (statement 2.1). Despite acceptable range in these two conditions changed between the two surveys (up to 1.5 x ULN in 2017 and up to 1.3 x ULN in 2023), this did not affect the consensus, corroborating the idea of a possible stricter IGF-1 target nowadays.

Overall, biochemical and clinical parameters should be more frequently checked in uncontrolled patients (statement 2.3) and should be integrated into the definition of resistance to fgSRLs (statement 3.1 and 3.3). In particular, a comprehensive evaluation of acromegaly, possibly through AcroDAT ® [26] and SAGIT® [27, 28], is considered valuable (statement 3.4). The absent or modest (< 20%) adenoma shrinkage is less relevant than biochemical measures in defining resistance (statement 3.2; agreement reached only in the second round); this may be due to the lack of evidence on a specific diameter/volume threshold to define clinically relevant shrinkage, particularly in small post-surgical remnants where MRI evaluation may be impacted by different artifacts [24]. Side-effects, such as hyperglycemia, should not be included in the definition of resistance to fgSRLs (statement 3.5), likely due to the reported marginal impact of fgSRLs on glucose homeostasis [29].

The possibility to predict resistance to fgSRLs would allow personalized interventions in patients identified at risk, avoiding long and ineffective attempts to control the disease [7, 11, 30]. Familiar history of pituitary adenomas and positive genetics are recognized as a predictive parameter for development of resistance (statement 4.1), particularly in young patients [31]. Absence of SSTRs and expression of molecular markers of aggressiveness in surgical pituitary tissue are strongly identified as markers of expected poor response to fgSRLs (statement 4.4–4.5). This is of great interest, since it implies that experts rely substantially on novel pathological techniques, recently found not to be routinely available also in Pituitary Tumor Centers of Excellence [32–34].

Finally, size of adenoma is not believed to be a predictor of resistance to fgSRLs (statement 4.2), while no consensus was reached on the role of elevated post-surgical GH/IGF-1 (statement 4.3); this is an interesting finding since virtually all guidelines report on the low likelihood of biochemical control in these two conditions. In the experience of part of the group, therefore, control could still be obtained in this context with fgSRLs. This opinion may be based on the long diagnostic delay in acromegaly, which is one of the main determinants of adenoma overgrowth and excessive GH and IGF-1 levels, with no implications on potential response to treatment [35, 36].

### Biochemical and clinical determinants for the choice of second-line therapy

In case of ineffective control of acromegaly with fgSRLs, combination therapy with cabergoline [11] did not meet the consensus as further therapeutic step for the panelists (statement 5.1), even if, recently, this combination showed a good IGF-1 normalization rate (30–58% of cases) [37]. Different factors may have contributed to the absence of consensus, including limited controlled studies, off-label use of cabergoline, availability of alternative therapeutic options, weak GH suppression by this drug and potential long-term detrimental effects (of heart valves especially in the context of acromegalic cardiopathy). Cabergoline is not perceived as valuable option also when looking at compliance (statement 10.1), a finding supported by a recent real-world analysis of US administrative claims data, which reported lowest adherence and persistence for this drug [30].

In line with guidelines, experts agree that PEGV monotherapy (statement 5.2), in combination with fgSRLs (statement 5.3), or PASI (statement 5.4) could be used in patients resistant to fgSRLs [5, 11]. Although PEGV in initial studies was only used as monotherapy [38, 39], ACROSTUDY real-world cohort showed that PEGV is used in up to 50% of cases in combination with fgSRLs [40], albeit with differences among countries [41].

Guidelines suggest that PEGV monotherapy is useful in patients with glucose metabolism disorders and/or without problems related to the pituitary mass [11]. The Delphi panel agreed that the presence of T2DM motivates the choice of PEGV as monotherapy (statement 6.3), with increased consensus compared to 2017 (86% vs 72%), indicating a better knowledge of this positive impact of PEGV [12, 42]. In resistant patients, the switch to PEGV monotherapy is motivated by slightly elevated or frankly elevated IGF-1 levels (statement 6.1 and 6.2), with a stronger agreement achieved in this second cohort of patients compared to patients with a lower level of elevation of IGF-1 (69% vs 86%).

Despite reported only in a minority of cases in clinical trials [43], the growth of pituitary mass upon fgSRLs discontinuation is a concern for switching to PEGV monotherapy (statement 7.1), albeit with a decreasing consensus compared to 2017 (69% vs 74%, respectively). Previous radiotherapy is a determinant for switching to PEGV monotherapy (statement 6.5), indicating that in this cohort of patients the concern for pituitary mass growth is reduced. This statement did not achieve a consensus in 2017, possibly indicating a growing reliability of new radiotherapy techniques in controlling tumor mass [44].

PEGV daily injections schedule (statement 7.3), and the possible reappearance of headache after SRLs discontinuation (statement 7.4) are also reasons for concern; however, concern on compliance achieved a borderline consensus, lower than in 2017 (67% vs 83% in 2023 vs 2017, respectively), and in a subsequent statement (10.3) panelists agreed on PEGV monotherapy (even if not daily) being a possible therapeutic strategy considering the patient's compliance and adherence (83%).

Despite being a drawback in 2017, therapy costs do not seem to be a detrimental factor for PEGV therapy anymore (statement 7.2, 9.3), likely in light of the significant efficacy demonstrated [45–47] on various targets including glycemia, presumably associated with a reduction of indirect costs. In fact, according to an Incremental Cost Effectiveness Ratio analysis in the Spanish NHS [48], PEGV is the most cost-effective alternative in the treatment of acromegaly in fgSRLs-resistant patients.

According to the panel, the transition to PASI should not necessarily take place after initial treatment with PEGV (either in monotherapy or combination, statement 8.1). This is in line with the 2018 consensus [11] but not with the most recent 2020 consensus, which positioned PEGV monotherapy as a second-line option, while fgSRLs + PEGV or PASI were reported as a third-line option [5]. Still, this is consistent with the European and Italian label for the drugs, which position them both as options after ineffectiveness of fgSRLs, and it is possibly related to some recent real-life data reporting an increased efficacy of PASI in fgSRLs-resistant patients [49] compared to initial studies [50].

The experts agree" on the use of PASI without particular concern in case of no medical history of T2DM (statement 8.3). Indeed, PASI could induce a worsening of the metabolic picture by inhibiting insulin and incretin secretion, especially in patients starting therapy with increased basal glycemia. No consensus was reached on the optimal treatment approach for the management of PASI-associated hyperglycemia (statement 8.4 and 8.5). Still, a multicenter, randomized, open-label, Phase IV study reported that, in some acromegalic patients treated with PASI, hyperglycemia could be effectively controlled by metformin, eventually followed by incretin-based therapy [51].

Real-life studies have also identified a discrete action of PASI on headache [52, 53], as acknowledged by the experts (statement 8.2). As per PEGV, costs are not a determinant for switching to PASI monotherapy (statement 9.4), while the panel show some cost-related concern for PASI + PEGV combination (statements 9.5). This is possibly related to the significant higher costs of this combination, associated with the panel’s lower expertise on this approach.

To summarize, the panelists show a clear preference within the Italian NHS for an independent decision-making process, encompassing nearly all individual or combined therapy schedules (statements 9.1–9.4). PASI monotherapy achieves the highest consensus for compliance and adherence compared to the other second-line medical treatment options (statement 10.2), while a consensus was not achieved on PASI + PEGV combination therapy (statement 10.5).

### Role of blood glucose levels in therapeutic management

Impaired glucose tolerance and T2DM are common in acromegaly [54] and guidelines suggest that T2DM should influence the choice of medical therapy. PASI is not recommended in uncontrolled T2DM patients because of the high risk of further glycemic control deterioration [4]; on the other hand, many data showed that PEGV treatment improves glucose metabolism [12, 43] and should be considered in patients with partial or no response to fgSRLs for whom glycemic control is challenging.

In the experts’ opinion, T2DM/metabolic alterations should be approached and treated as in the general population (statement 13.4, 13.5). Hyperglycemia onset should not modify acromegaly therapy, even if induced by it (statement 12.1). An HbA1c target below 7.0%, or further tailored to the patient’s age (statement 12.2, 12.4) and the management of cardiovascular risk factors (statement 12.6) are goals for acromegaly and hyperglycemia treatments. In the second round, the panel reached a consensus on a lower HbA1c target (below 6.5%, statement 12.3), a parameter that sparked disagreement in 2017. This finding, in line with the most recent T2DM guidelines [55], is possibly due to more efficacious and safer treatment options for diabetic patients, while a more stringent diabetes control is probably also seen as a proactive measure to mitigate cardiovascular mortality.

No consensus was reached on the referral of patients to a Diabetology Centre (statement 12.5), likely reflecting the different organization of Italian Endocrinology practices, where only some centers have Diabetology Units. It could be assumed that some neuroendocrinologists personally manage T2DM to avoid delocalization of patients, while others, considering acromegaly diabetes as non-specific form of diabetes, prefer referring these patients to diabetologists.

In fgSRL resistant patients, the choice of second-line depends on metabolic compensation (statement 14.1), but a consensus could not be reached on whether diabetes mellitus per se should influence the second-line medical therapy (statement 14.2) and on the addition of PEGV in these patients (statement 14.3). On the other hand, panelists do not perceive that in these cases the only possibility to start PASI is in association with PEGV (statement 14.4), probably also considering that PASI’s detrimental effect on glucose metabolism is only partially counteracted by PEGV [56]. Experts did not agree on IFG influencing the choice of second-line medical therapy, contraindicating, at least in young patients, the use of PASI (statement 11.1), or indicating the necessity to switch to PEGV monotherapy (statement 11.4). This is an interesting finding, since data from literature show that patients with IFG are at higher risk of developing hyperglycemia when switching to PASI [57], while age seems to be protective against development of T2DM while on this therapy.

FgSRLs are not considered responsible for metabolic worsening, or at least not such as to disregard their effectiveness on biochemical or tumor mass control. The onset of T2DM in well-controlled patients is not a valid reason for fgSRLs therapy discontinuation (statement 13.1), which should be accompanied by the start of an antidiabetic drug (statement 13.2). This despite evidence demonstrating significant positive impact on glucose metabolism of PEGV, regardless of IGF-1 levels [43]. Therefore, in the experts’ opinion the attainment of effective control over acromegaly is the priority. The positive effects on GH/IGF-1 secretion and tumor mass are deemed more crucial, with any potential deterioration in glycemic control being considered a separate concern. It is noteworthy that these assertions achieved a more robust consensus than in 2017. However, when T2DM treatment is ineffective, the experts agree on a switch to PEGV monotherapy also in well-controlled patients (statement 13.3); this statement did not achieve a consensus in 2017, indicating that, at least in this subpopulation, the panelists agreed that the positive effect of PEGV on glucose metabolism may be exploited to manage both conditions.

Experts agree that in a patient with history of GDM and a GH-secreting macroadenoma resistant to fgSRLs, surgical intervention should be prioritized (statement 15.4). Another option is the combination of fgSRLs and PEGV (considering the diabetogenic and tumor growth risks if fgSRLs are discontinued and PEGV is used as monotherapy) (statement 15.2). An alternative that gains consensus in the second round is shifting to PASI (prioritizing the tumor mass issue over the diabetes concern, statement 15.1).

### Limitations

This work presents the limitations of the Delphi studies: a decline in response rate between the rounds of 17% was observed, but this is within the limits described for Delphi studies, especially when dealing with a large number of statements [58, 59]. The consistency of answers in subsequent interactions indicated that the statements were correctly defined. Although some of the invited experts did not participate, those taking part well represented the real-world management of acromegaly in Italy. The lack of consensus for some statements underlines the open questions that need further research to be addressed.

## Conclusions

**Fig. 3:**
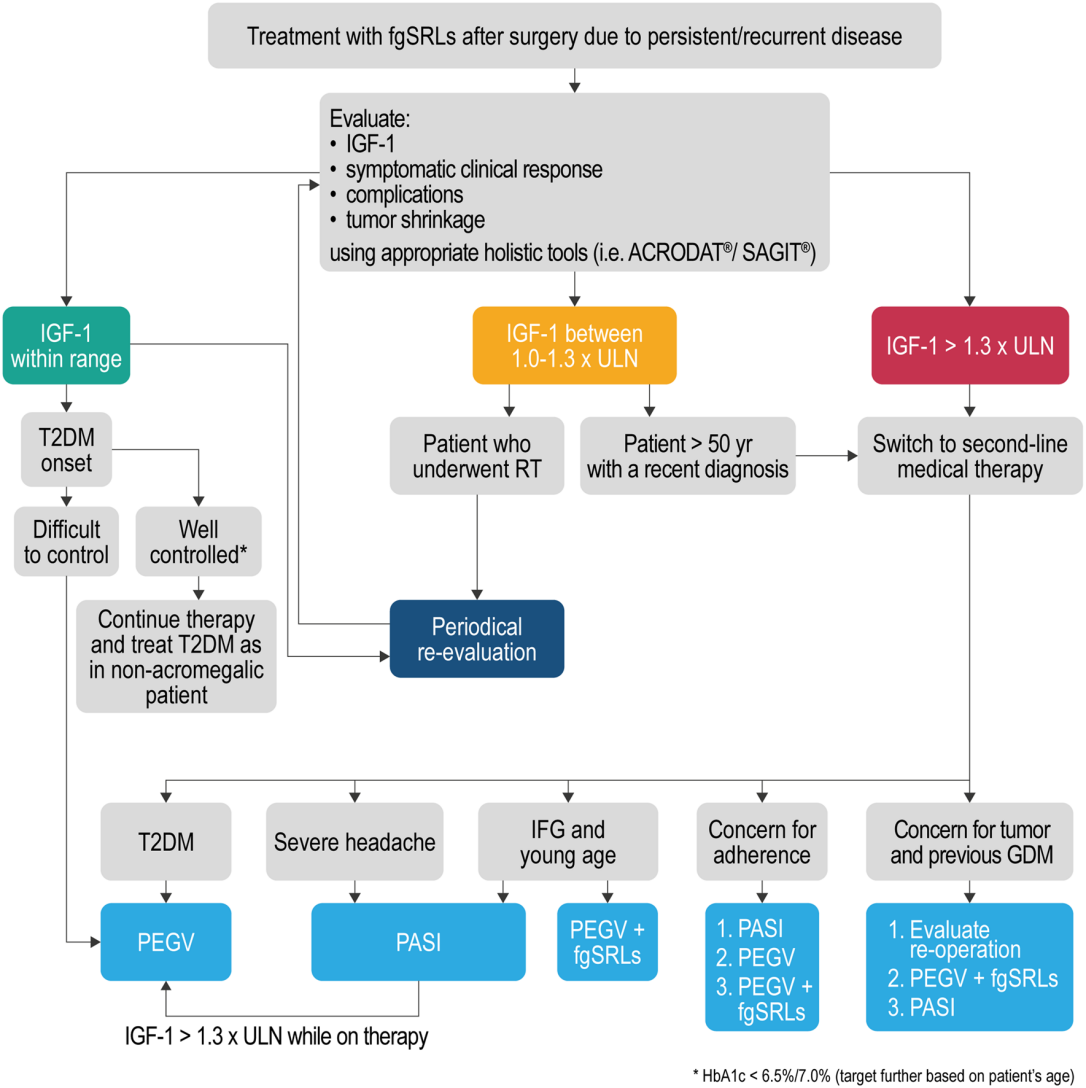
Algorithm summarizing a possible clinical management of medical therapy of acromegaly based on the opinion of the Italian expert panel

A summary of the possible management of acromegalic patient resistant to first-line medical treatment based on what emerged from the answers provided by the Delphi panel is reported in Fig. 3.

The experts agreed on a holistic management approach to acromegaly, considering IGF-1 levels (with more stringent targets than in the past, at least in some categories of patients), tumor mass, complications, symptoms, and the patient’s QoL. This aligns with current guidelines, which emphasize the importance of incorporating CROs/PROs.

Hyperglycemia is determinant for second-line medical therapy choice. PEGV positive effect on glucose metabolism is not always valued, especially in well controlled patients with fgSRLs who develop glucose derangement. On the other hand, concern about the diabetogenic effect of PASI seems to be somehow attenuated, especially in young patients, and no consensus on its management was achieved. T2DM should be managed as in non-acromegalic patients, but with more strict targets than in the past, possibly due to the known cardiovascular risk of this population, further amplified by diabetes [4]. The direct cost of therapy is of minor importance likely because of a greater attention to the indirect costs associated with the complications, which increase with uncontrolled disease. It is therefore necessary to choose currently available highly effective second-line medical treatment (PEGV and PASI) based on the characteristics of the patients.

## Supplementary Information

Below is the link to the electronic supplementary material.

Supplementary file1 (DOCX 180 KB)

## Data Availability

Data available within the article or its supplementary materials.
