# Supplementary material for: Insights from an Italian Delphi panel: exploring resistance to first-generation somatostatin receptor ligands and guiding second-line medical therapies in acromegaly management
Source: J Endocrinol Invest. 2024 May 29;47(12):2999–3017. doi: 10.1007/s40618-024-02386-3 (PMC11549125; doi:10.1007/s40618-024-02386-3)
Supplement: Supplementary file 1 — Supplementary file1 (DOCX 180 KB) [file 40618_2024_2386_MOESM1_ESM.docx]

**Insights from an Italian Delphi panel: exploring resistance to first-generation somatostatin receptor ligands and guiding second-line medical therapies in acromegaly management**

Silvia Grottoli, Pietro Maffei, Alberto Stefano Tresoldi, Simona Granato, Laura Benedan, Paolo Mariani, Andrea Giustina

**Methods**

*The Delphi method*

The Delphi method is a widely employed consensus approach in clinical research, particularly when addressing complex or uncertain topics. It involves multiple rounds of anonymous surveys or questionnaires that allow experts to provide feedback and revise their responses based on the collective insights of the group. The method facilitates the achievement of a consensus among experts and results in well-informed decisions and recommendations regarding clinical studies (1). This methodology has demonstrated its effectiveness in bridging knowledge gaps related to rare diseases (2, 3) and has proven valuable in providing local guidance for the management of acromegaly (4).

The fundamental prerequisites of a high-quality Delphi study are anonymity, iteration, controlled feedback, and statistical stability of consensus (5).

*Definition of the statements*

After an extensive literature review, the authors identified key topics for the analysis and crafted related statements during a series of online meetings held in June and July 2023. These discussions included the evaluation of statement proposed in a previous Delphi study conducted in 2017 that was not published. Statements were discussed and modified to ensure their alignment with the most recent clinical developments and were integrated into an online questionnaire, approved from the Steering Committee (AG, Silvia G, and Pietro M).

*Delphi questionnaires*

*Delphi Questionnaire 2023*

The questionnaire encompassed a total of 75 statements.

Each statement was rated on a 6-point Likert scale. The ratings were defined as follows:

1 = Strongly Disagree

2 = Disagree

3 = Slightly Disagree

4 = Slightly Agree

5 = Agree

6 = Strongly Agree

A neutral option was omitted to encourage respondents to take a clear position in terms of agreement or disagreement, albeit with varying degrees.

In the second round, two statements were slightly reviewed before resubmission to the expert panel, to eliminate any potential ambiguities or misinterpretations. The rephrasing was as follows for statement 1.3: round 1, “IGF-1 levels within the normal range represent an important goal of therapy, but they can be considered satisfactory even if they remain slightly above the normal range”; round 2, “IGF-1 levels within the normal range represent an important goal of therapy, but they can be considered satisfactory even if they remain slightly above the normal range (1.0 – 1.3 x ULN)”.

For the statement 2.5: round 1, “In case of discrepancy between normalization of IGF-1 and GH, to define acromegaly as controlled, both values need to fall within the target range”; round 2, “In case of discrepancy between normalization of IGF-1 and GH, the disease cannot be considered controlled as I believe control is achieved only when both parameters are normalized”.

*Delphi Questionnaire 2017*

The questionnaire evaluated in 2017 encompassed 61 statements organized in the following topics: SRLs therapy, resistance to SRLs, second-line choice, evaluation of hyperglycemia and comorbidities in a patient with acromegaly. Each statement was rated on a 5-point Likert scale. The ratings were defined as follows:

1 = Fully Disagree

2 = Disagree

3 = Agree

4 = More than agree

5 = Fully Agree

Percentage of disagreement was calculated including points 1 and 2. Percentage of agreement was obtained from points 3, 4 and 5.

The statements evaluated were the following:

| 1. I believe that in the evaluation of resistance to somatostatin receptor ligands (SRLs): | |
| --- | --- |
| 1.1 | IGF-1 levels represent the "gold standard" for assessing resistance to SRLs |
| 1.2 | To assess resistance to SRLs it is necessary to repeat the IGF-1 dosage several times over time, especially when its increase is moderate |
| 1.3 | IGF-1 levels within the normal range represent an important goal of therapy, but they can be considered satisfactory even if they remain slightly above normal |
| 1.4 | Both IGF-1 and GH levels are essential in defining a condition of resistance to SRLs |
| 1.5 | In my experience, IGF-1 measurements exhibit limited or inconsistent reliability, and therefore GH levels should continue to be used to decide whether the patient is resistant to SRLs therapy |
| 1. I believe that to contextualize the role of biochemical parameters in assessing resistance to SRLs: | |
| 2.1 | Slightly increased IGF-1 levels (1.0-1.5 x ULN) during SRLs therapy could be attributable to a quantitatively excessive or qualitatively unbalanced caloric intake rather than to real drug resistance |
| 2.2 | Slightly increased IGF-1 levels (1.0-1.5 x ULN) during SRLs therapy are tolerable in a patient over 50 with a recent diagnosis, as in their case, acromegaly could be less aggressive |
| 2.3 | Slightly increased IGF-1 levels (1.0-1.5 x ULN) during SRLs therapy are tolerable in a woman with preserved gonadal function because estrogens play a protective role |
| 2.4 | Slightly increased IGF-1 levels (1.0-1.5 x ULN) during SRLs therapy are tolerable in a patient who has already undergone stereotactic radiosurgery as a progressive improvement in disease control is expected |
| 2.5 | In patients who do not achieve optimal disease control during SRLs therapy, GH and IGF-1 measurements, as well as clinical assessments should be repeated more frequently |
| 1. I believe that therapy with SRLs: | |
| 3.1 | Reduces tumor volume in approximately 50% of cases |
| 3.2 | Determines tumor volume control only when used as a first-line therapy |
| 3.3 | To be considered effective, it must cause a reduction of more than 50% in tumor volume |
| 3.4 | When not effective in reducing tumor volume with conventional analogues, the use of second-generation analogues should be considered |
| 3.5 | In patients not biochemically controlled and with an invasive macroadenoma, should be recommended in association with pegvisomant |
| 1. Given that SRLs reduce the size of the GH-secreting adenoma relatively frequently, in clinical practice and before a possible surgical approach I believe this is an objective: | |
| 4.1 | primary in all patients with macroadenoma |
| 4.2 | primary, but only in the presence of a suprasellar macroadenoma |
| 4.3 | primary, because the normalization of biochemical markers parallels a reduction of tumor volume |
| 4.4 | secondary, because the primary objective is the normalization of hormonal markers |
| 4.5 | secondary, because in the majority of patients it has no clinical significance |
| 1. I believe that in the patient not controlled by SRLs, the second-line therapeutic choice has a specific effect, independent of hormonal normalization, on: | |
| 5.1 | QoL |
| 5.2 | arthropathy and fracture risk |
| 5.3 | cardiovascular damage |
| 5.4 | obstructive sleep apnea syndrome |
| 5.5 | no specific effect on QoL, arthropathy/fracture risk, cardiovascular damage/sleep apnea |
| 1. I believe that the following therapeutic goals should be pursued in the acromegalic patient who develops hyperglycemia, with the following monitoring methods:   (it is appropriate to underline that statement 6 concerns the acromegalic patient tout court and not only those eligible for second-line medical treatment) | |
| 6.1 | An HbA1c level below 7.0% with the same frequency and modalities of the non-acromegalic diabetic patient (e.g., every 3-6 months based on the degree of compensation) |
| 6.2 | A more ambitious HbA1c level (e.g., less than 6.5%) with greater frequency and use of glycemic self-monitoring compared to non-acromegalic diabetic patients |
| 6.3 | A less ambitious HbA1c level (e.g., lower than 7.5-8.0%) with less frequency and use of glycemic self-monitoring compared to non-acromegalic diabetic patients |
| 6.4 | A fasting blood glucose value between 90 and 130 mg/dl and a postprandial <180 mg/dl in addition to an HbA1c value of less than 7% |
| 6.5 | A less ambitious HbA1c level (e.g., lower than 7.5-8.0%), but with intensification of control of non-glycemic cardiovascular risk factors (blood pressure, lipids, body weight) |
| 1. In a well-controlled acromegalic patient undergoing therapy with SRLs who develops diabetes mellitus, I believe that: | |
| 7.1 | SRLs dose should be reduced or the SRL therapy should be discontinued, and treatment with pegvisomant should be initiated |
| 7.2 | Pegvisomant treatment should be associated to SRLs therapy |
| 7.3 | SRLs therapy should be maintained, and an anti-hyperglycemic pharmacological treatment should be initiated |
| 7.4 | SRLs therapy should be discontinued, and treatment with pegvisomant should be initiated only if hyperglycemia cannot be managed with antidiabetic medications |
| 7.5 | The decision on therapy should be postponed until the patient remains within acceptable levels of glycemic control (e.g., fasting blood sugar < 180 mg/dl, HbA1c < 7.0-7.5%) |
| 1. In a 55-year-old male acromegalic patient with endo-suprasellar macroadenoma, sleep apnea and type 2 diabetes, I consider the disease controlled if: | |
| 8.1 | Normalization of biochemical parameters has been achieved (normalization of IGF-1 and random GH levels < 1.0 g/L) |
| 8.2 | The improvement of subjective symptoms has been achieved as well as the normalization of IGF-1 and random GH levels < 1.0 g/L |
| 8.3 | The normalization of the biochemical parameters (normalization of IGF-1 and random GH levels < 1.0 g/L), the improvement of the metabolic and respiratory picture, the reduction of the size of the tumor or its stabilization have been achieved |
| 8.4 | Normalization of biochemical parameters (normalization of IGF-1 and random GH levels <1.0 g/L) and reduction of tumor size have been achieved |
| 1. In an acromegalic patient treated with somatostatin analogues, I believe the disease is NOT controlled: | |
| 9.1 | When, even if tumor shrinkage is not achieved, normalization of biochemical parameters is observed |
| 9.2 | When tumor shrinkage is achieved despite of failure to normalize biochemical parameters |
| 9.3 | When an increase in tumor volume occurs despite of normalization of biochemical parameters |
| 9.4 | When stabilization of the tumor mass is achieved despite of failure to normalize the biochemical parameters |
| 1. In reference to the weight to be given to arthropathy in defining acromegalic disease as controlled in a patient with multiple comorbidities: | |
| 10.1 | I would not give any weight to the arthropathy if the biochemical parameters were normal and the other complications of the disease had improved |
| 10.2 | Although it has an important impact on the quality of life, I do not consider it relevant in the definition of disease control as it cannot be modified by therapy |
| 10.3 | The improvement of the joint condition is part of the criteria I use to define the disease as controlled |
| 10.4 | Arthropathy is a disabling condition and therefore I consider its control one of the key criteria, in addition to the normalization of biochemical parameters, to define acromegalic disease as controlled |
| 1. I believe that the motivating factor for shifting to pegvisomant monotherapy in a patient resistant to SRLs could be represented by: | |
| 11.1 | Elevated IGF-1 in the range 1.1-1.5 x ULN (during SRLs therapy) |
| 11.2 | Elevated IGF-1 > 1.5 x ULN (during SRLs therapy) |
| 11.3 | The presence of diabetes |
| 11.4 | Cardiovascular complications |
| 11.5 | A history of radiotherapy |
| 1. In the decision to shift to pegvisomant monotherapy in a patient resistant to SRLs, the following can be a cause for concern: | |
| 12.1 | The regrowth of the pituitary adenoma upon SRLs discontinuation |
| 12.2 | The therapy costs |
| 12.3 | The patient's compliance when transitioning to daily therapy |
| 12.4 | The recurrence of headache in a patient for whom this symptom was responsive to SRLs therapy |
| 12.5 | I do not see any specific reason for concern |
| 1. I believe it is appropriate to make a shift to pegvisomant as monotherapy in patients resistant to SRLs: | |
| 13.1 | After 6 months of SRLs treatment |
| 13.2 | After 12 months of treatment with SRLs treatment |
| 13.3 | After 24 months of SRLs treatment |
| 13.4 | Never, because I do not think it is appropriate to shift to pegvisomant as monotherapy |

**References**

1. Hasson F, Keeney S, McKenna H. Research guidelines for the Delphi survey technique. J Adv Nurs. 2000;32(4):1008-15.

2. Pivonello R, Scaroni C, Polistena B, Migliore A, Giustina A. Unmet needs on the current medical management of Cushing's syndrome: results from a Delphi panel of Italian endocrinologists. J Endocrinol Invest. 2023;46(9):1923-34.

3. Scarpa M, Barbato A, Bisconti A, Burlina A, Concolino D, Deodato F, et al. Acid sphingomyelinase deficiency (ASMD): addressing knowledge gaps in unmet needs and patient journey in Italy-a Delphi consensus. Intern Emerg Med. 2023;18(3):831-42.

4. de Pablos-Velasco P, Venegas EM, Alvarez Escola C, Fajardo C, de Miguel P, Gonzalez N, et al. Diagnosis, treatment and follow-up of patients with acromegaly in a clinical practice setting in Spain: the ACROPRAXIS program Delphi survey. Pituitary. 2020;23(2):129-39.

5. Nasa P, Jain R, Juneja D. Delphi methodology in healthcare research: How to decide its appropriateness. World J Methodol. 2021;11(4):116-29.
